# Liquid Biopsy: The Unique Test for Chasing the Genetics of Solid Tumors

**DOI:** 10.1177/2516865720904052

**Published:** 2020-02-28

**Authors:** Seyed Mohammad Kazem Aghamir, Ramin Heshmat, Mehdi Ebrahimi, Fatemeh Khatami

**Affiliations:** 1 Urology Research Center (URC), Sina Hospital, Tehran University of Medical Sciences, Tehran, Iran; 2 Chronic Diseases Research Center, Endocrinology and Metabolism Population Sciences Institute, Tehran University of Medical Sciences, Tehran, Iran; 3 Department of Internal Medicine, Faculty of Medicine, Sina Hospital, Tehran University of Medical Sciences, Tehran Iran

**Keywords:** Liquid biopsy, cell-free nucleic acids (cfNAs), circulating tumor cells (CTCs), exosomes

## Abstract

Blood test is a kind of liquid biopsy that checks cancer cells or cancer nucleic acids circulating freely from cells in the blood. A liquid biopsy may be used to distinguish cancer at early stages and it could be a game-changer for both cancer diagnosis and prognosis strategies. Liquid biopsy tests consider several tumor components, such as DNA, RNA, proteins, and the tiny vesicles originating from tumor cells. Actually, liquid biopsy signifies the genetic alterations of tumors through nucleic acids or cells in various body fluids, including blood, urine, cerebrospinal fluid, or saliva in a noninvasive manner. In this review, we present an overall description of liquid biopsy in which circulating tumor cells, cell-free nucleic acids, exosomes, and extrachromosomal circular DNA are included.

## Introduction

Cancer is a major worldwide health problem that originates from abnormal and uncontrolled cell division and can eventually spread into other tissues.^1^ In the past, it was wrongly thought that tumor genes and cells are only present in the exact tumor site. In 1896, the suggestion that circulating tumor cells (CTCs) are a major requirement to metastasis was first proposed by an Australian pathologist, Thomas Ashworth.^2^ The presence of cell-free DNA (cfDNA) was reported in human plasma by Mandel and Metals in 1948.^3^ Unfortunately, the concept of liquid biopsy was totally ignored till 1977, when researchers made the novel discovery that cancer patients carried cell-free nucleic acids (cfNAs) and cells in their peripheral blood.^4^

The exact word of “liquid biopsy” is classically applied for cfDNA and CTCs, but its component can be more. In fact it is a diagnostic molecular test to run on a sample of plasma to discover cancer cells that are shedding from primary or metastatic tumors that are circulating in the blood or DNA fragments of tumor cells.^5^ Tissue biopsy is a snapshot of tumor and cannot cover tumor heterogeneity.^6^ Liquid biopsy can be beneficial for early diagnosis of cancer or to examine the treatment efficacy, and because of its noninvasive nature, several samplings of blood over time are possible.^7-9^

**Figure 1.**
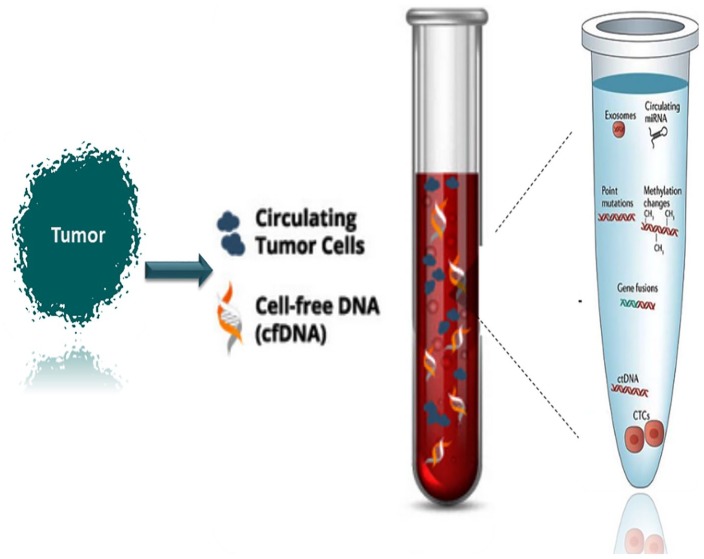
Schematic figure of the liquid biopsy and the way that its genetic components are presented in the blood.

In this review, we explain the molecular importance of the CTCs, cfNAs, and exosomes as the main component of liquid biopsy, in addition to the extrachromosomal circular DNA (eccDNA) as a possible component of liquid biopsy (Figure 1).

## Advantages of Liquid Biopsy

In the field of cancer management, there are some problematic issues concerning the early diagnosis, prognosis, and prediction of treatment resistance.^10-12^ Tumor heterogeneity brings a lot of difficulties for clinicians and stand in the way of cancer treatment.^13^ The molecular genetics profile of a tumor changes over time and standard tissue biopsies are able to represent an overall view of the tumor.^14-16^ Moreover, tissue biopsy is an invasive procedure and so is not easily repeatable considering the cost and the risk factors.^17^ Traditionally, routine histological evaluation and immunohistochemical study have an essential role in several aspects of diagnosis in both neoplastic and non-neoplastic disorders.

Currently, liquid biopsy, as a real-time representative of tumor, is a minimally invasive biopsy method which has been studied by scientists and oncologists over the past decades.^9^,^18^,^19^ The purpose of liquid biopsy is to identify and examine the biological material circulating in body fluid, originating within and from the tumor.^20^,^21^ Several research works have defined certain benefits of molecular information about primary tumors by using liquid biopsies and compared the result of liquid biopsy with tumor biopsy.^22-30^ There are controversies that liquid biopsy can take the place of tissue biopsy or will support “gold standard” tissue biopsies. According to Miro Venturi, Roche’s global head of diagnostics biomarkers, “Liquid biopsies could be a game-changer in cancer testing.” In fact, the minimally invasive nature of liquid biopsy brings a new insight for malignancy checking without delay, expenses, and risks, possibly at a microscopic stage, before the radiological tests.^31^ Liquid biopsy promises early detection of cancer because of its potential for detecting a classically incurable malignancy at an earlier, more curable, and even curable stage.^32^ The use of circulating tumor DNA (ctDNA) genetic changes within blood test has extraordinary applications for the period of cancer treatment, with dynamic monitoring of therapy response, quick detection of resistance, and foretelling the tumor recurrence ahead of clinical relapse.^33-35^

## Circulating Tumor Cells

Circulating tumor cells are tumor cells that are driven from the primary tumor and are released into the blood vessels or lymphatic vessels.^36^ The existence of CTCs in the blood of metastatic prostate cancer patients was reported by Thomas Ashworth, an Australian physician, in 1869.^2^ Metastasis is the most challenging process of tumor and the key cause of cancer-associated death (9 of 10 deaths). The “seed and soil” hypothesis described that tumor invasion—the intrinsic characteristics of the tumor cells (seeds) and host microenvironment (soil)—are the central elements of the place of tumor development.^37^ Watanabe in 1954 by injecting bronchogenic carcinoma cells of mice showed that the CTCs can be the key role players in metastasis.^38^ It was shown that the metastases development, to some extent, depends on the size and quantity of the CTC clusters.^39^,^40^ The CTCs are highly heterogenic, and several types of CTCs are suggested, which are given below:

*Traditional CTCs*: The hallmark of these cells is having large and irregular shapes, with an intact, viable nucleus; they express cytokeratins with epithelial origin and not hematopoietic origin (they do not have CD45).^41^,^42^
*Cytokeratin-negative (CK*^−^*) CTCs*: These types of CTCs are cancer stem cells passing through the epithelial-mesenchymal transition steps and forming the cytokeratin-negative CTCs. They can be opposed to treatment and is the most potential one to invade and form metastasis because they do not have cytokeratins and CD45.^43-45^
*Apoptotic CTCs*: These are the traditional CTCs that undergo apoptosis. They are distinguishable with nuclear fragmentation or cytoplasmic blabbing associated with cell death. The proportion of apoptotic CTCs compared with the traditional CTCs can be a predictor of treatment efficacy.^46^,^47^
*Small CTCs*: These are recognized as the cytokeratin-positive and CD45-negative cells, similar in size to leukocytes. Notably, the small CTCs have been concerned with progressive disease and differentiation into small cell carcinomas, which often need special therapeutic strategies.^48^,^49^

However, the new classification system suggested that CTCs should be characterized by their size and the expression of several markers such as EpCAM.^50^ The principle of CTC isolation, quantification, and characterization is based on the different physical characteristics (magnitude, electric charges) and genetic properties of the CTCs compared with the nontumor cells.^51^,^52^ Isolation by size of the epithelial tumor cell (ISET) platform is used for CTC cluster isolation based on tumor and nontumor cell differences.^53^ Interestingly, physical property can also support micro-fluidic technology, which results in making a new flexible micro-spring array device.^54-57^ The CellSearch platform, Menarini Silicon Biosystems (https://www.cellsearchctc.com/) system, which is based on EpCAM- and cytokeratin-positive CTC selection, is the only Food and Drug Administration (FDA)–approved one and is used in numerous clinical studies.^58-61^ In breast cancer, it was found that metastatic breast cancer patients who had CTCs ⩾5, contrary to patients with CTCs <5, in 7.5 mL of blood had failed first-line treatment.^62-64^ The cutoff value (CTCs ⩾5/7.5 mL of blood) in prostate cancer highlighted the fact that the higher CTC numbers resulted in poorer overall survival.^65^,^66^ The new cutoff is a CTC threshold of 5 cells per 7.5 mL, which means patient with CTCs ⩾5 were classified as Stage IV_aggressive_ and those with CTCs <5 as Stage IV_indolent_.^67^ In colorectal cancer, the comparable predictive value of the CTC counts was optional for the selection of first-line treatment.^68^,^69^ Higher quantity of CTC number was linked to lower survival in lung cancer as well.

## Cell-Free Nucleic Acids

“Cell-free nucleic acids” is a general term and comprises cfDNA, cell-free RNA (cfRNA), and cell-free mitochondrial DNA (cf-mtDNA). Cell-free DNA are nonencapsulated DNA, free from cells in the bloodstream. They originate from a tumor clone.^70^ In eukaryotic nucleus, the DNA exists in a structured form termed nucleosomes which consist of around 170 base pairs of DNA wrapped around a core histone octamer (H2A, H2B, H3, and H4), linked by 10 to 100 base pairs of naked DNA as a linker DNA.^71^,^72^ The fragmented DNA, in the form of nucleosomes, can be released by various tumor cells, and then these enter the bloodstream during apoptosis or necrosis and normally are removed with macrophages.^73^,^74^ In cancerous condition, these cfDNA fragments are overproduced by the tumor cells, which are left behind that the macrophages cannot clean up completely.^75^ The first connection between cancer and elevated cfDNA was shown in 1977 by Leon et al.^76^ Then, Stroun et al^77^ established this link by separating DNA gained from the plasma of cancer patients and analyzing them. Patients with malignant epithelial gastrointestinal tumors have more cfDNA in comparison with patients with benign disease.^78^ The cfDNA is typically present in plasma as a double-stranded form (dsDNA), even though the single-stranded form can also been recognized.^79^,^80^

In 1987, the circulating cfRNA, in the form of RNA proteo-lipid complex, was isolated from the serum of malignant ones.^70^,^81^ A cfRNA scan of messenger RNA and microRNA (miRNA) is made. In 1999, the presence of cfRNAs was established in the plasma or serum of patients with nasopharyngeal carcinoma and malignant melanoma.^82-84^ Moreover, the presence of cfRNA was proven in patients with breast cancer, colorectal cancer, follicular lymphoma, and hepatocellular carcinoma.^85-87^ The circulating cell-free circulating microRNAs are the new course of biomarkers as they possess all the crucial features such as sensitivity, predictability, specificity, robustness, translatability, and noninvasiveness. MicroRNAs are small noncoding RNAs that act as suppressors of protein translation and disturb the protein expression panel at posttranscriptional mechanisms.^88^

Thanks to the latest advances in molecular genetics techniques, taking the genetic and epigenetic alterations of cfDNA and cfRNA into consideration is not very hard.^89^,^90^ Several studies on “Catalogue of Somatic Mutations in Cancer” (COSMIC) mutations, using next-generation sequencing (NGS), verified that circulating cfDNA analysis can be an excellent prognostic and diagnostic tool.^91-93^ One of the most promising applications of ctDNA is treatment response monitoring.^94-96^ It is also suggested that ctDNA-based analysis possibly will develop the controlling of patients with possibly treatable or metastatic disease.^97^ Recently, Newman et al. and Murtaza M et al.^98^,^99^ have suggested an ultrasensitive and cost-effective method entitled cancer-personalized profiling by deep sequencing for cfDNA quantification.

Mainly, DNA methylation in CpG islands as the epigenetic event happens nearly at the beginning of cancer development, and so it has the potential of being a biomarker for early diagnosis.^100-102^ So, DNA methylation of cfDNA can be checked and several markers can be proposed, including both global genomic hypomethylation (Alu elements) and gene-specific methylation such as *GSTP1* methylation in prostate cancer, methylation of cfDNA in *SEPT9* promoter region in colorectal cancer, and *RASSF1A* in different cancer types.^103^,^104^ It is shown that genome-wide cfDNA methylation profiles are extremely counterpart with detected methylation in corresponding tumor tissues.^105^ The methylated cfDNA biomarkers are a comprehensive noninvasive monitoring tool of treatment response in metastatic colorectal cancer.^106^,^107^ SOX17 promoter methylation in CTCs and matched cfDNA isolated from plasma of patients with breast cancer indicated a direct connection between the presence of CTCs and cfDNA in patients with operable breast cancer, after surgical removal of the primary tumor.^108^

## Extrachromosomal Circular DNA

In the 1980s, the presence of endogenous DNA circles originating from canonical linear chromosomal loci, identified as eccDNA, was described in nuclear fractions of plant cells (wheat and tobacco).^109^ In fact, the main machinery of oncogenes to aggregate their copy number occurs by eccDNA.^110^ It was shown that eccDNA can be seen in approximately half of human cancers, while its frequency is different by tumor type.^111^,^112^ The presence of tumor eccDNA in blood as a liquid biopsy component has been suggested very recently.^113^

## Exosomes

Microvesicles and exosomes, collectively referred to as extracellular vesicles (EVs), are lipid bilayer structure vesicles that are released from all eukaryotic cells and play an important role in the instruction of extracellular communication, cellular differentiation, cell migration, and maintenance of normal tissue condition.^114^ The size of the exosomes varies from 30 to 100 nm, and they are secreted through the interior budding of the plasma cell membrane.^115^ The exosomes can be released through both normal (epithelial, mesenchymal, and immune) and cancerous cells in different settings such as blood, urine, and sputum.^116^ They were first described by Pan and Johnstone in 1983 at McGill University.^117^ It was suggested that there is a connection among the existence of cfNAs in plasma and exosomes because one possible mechanism for the release of the cfNAs into blood is by exosomes.^118^,^119^ The transferring of genetic information from the exosomes to the host cells (receiver of exosomes) is possibly involved in the metastatic conversion of the host/receiver cells.^120^ Exosomes of diverse cell types have unlike proteins that can be potentially used as biomarkers in clinical experiments.^121^

Exosomes contain dsDNA of the parent cell, so they could be released from a specific tissue or from a specific tumor via the exosomal surface biomarkers.^122^,^123^ Using sensitive detection technologies such as nano-particle tracking analysis (ZetaView), Western blotting techniques, transmission electron microscopy, the Agilent Bioanalyzer system, and modern droplet digital polymerase chain reaction techniques, we are able to assess the exosomal nucleic acids.^124^,^125^

Exosome-based liquid biopsy in comparison with the CTCs and cfNAs are more homogeneous in terms of size.^126^ Many isolation and characterization protocols are established to prepare the exosomes for the diagnosis of cancer and its therapy.^126^,^127^

## Clinical Applications of Liquid Biopsy

In fact, CTC, ctDNA, and exosomes have broad biomarker potential because they can timely and dynamically represent the tumor’s genetic status both for diagnosis and for prognosis applications. It was suggested that liquid biopsy has a better sensitivity and is extra convenient as a cancer diagnosis tool in comparison with the traditional tissue biopsy methods.^128^ In the middle of 2016, the first liquid biopsy test was approved by the FDA.^129^,^130^ The point mutation of exon 19 deletion or exon 21 [L858R] in the *epidermal growth factor receptor* (*EGFR*) gene of ctDNA was approved as a good predictor of the response to the EGFR tyrosine kinase inhibitors in non–small-cell lung cancer patients.^131^,^132^ When the first test of ctDNA was approved, several studies had already shown the impact of liquid biopsy in the field of cancer management. Several studies have stated that breast and ovarian cancers with ctDNA microsatellite instability have deprived prognosis.^133-135^ The presence of apoptotic CTCs or fragmented CTCs in the peripheral blood can be an indicator of cancer.^24^,^136^ Although imaging and tissue biopsy are still the gold standards in solid tumor screening and monitoring, prospective studies have suggested that CTC detection with imaging combination is the greatest choice.^137^,^138^ The quick diagnostic value of CTCs in primary stages of cancer has been considered several times, and in an animal model, it was shown that CTCs were established very quickly in the “carcinoma in situ” stage, implying the fact that the tumor cells had spread prior to diagnosis.^139^,^140^ The exceeding indication showed that the CTCs may be a valuable tool for very early cancer diagnosis. The detection of CTCs in different cancer types including colorectal cancer, lung cancer, and prostate cancer correlates with different pathological stages, clinical outcome, and patient’s survival.^141-146^ The first and only actionable test for detecting CTCs in cancer patients is the CellSearch system, which is applicable as a self-determining forecaster of overall and progression-free survival in metastatic breast, prostate, and colorectal cancer.^147^,^148^ In addition to CTC detection, ctDNA is a gifted approach for primary cancer diagnosis. Many researchers have indicated a positive connection of ctDNA quantity and tumor stage to predict the severity of malignancies and efficacy of treatment.^90^,^149^,^150^ The use of exosomes as a predictive biomarker and indicator of treatment response completely relies on its protein and miRNA expression profiles. It was shown that the downregulation of exosomal miR-92a in hepatocellular carcinoma was connected to cancer development and a high risk of relapse, and the overexpression of exosomal miR-21-3p is a sign of cisplatin resistance in ovarian cancer.^151^,^152^

At MD Anderson Cancer Center, the role of liquid biopsy as a medical tool for patients with papillary thyroid carcinoma (PTC), medullary thyroid carcinoma (MTC), and anaplastic thyroid carcinoma is analyzed. In patients with PTC and MTC, it is shown that the concordance between tissue and liquid biopsy was more than 80%.^153^

Thanks to the latest advances in molecular technology detection, in methods such as NGS, whole-genome shotgun bisulfate sequencing, and tagged-amplicon deep sequencing, day by day, the importance of liquid biopsy is highlighted more and more. Most scientists believe that its use in cancer treatment will support the conventional tissue biopsy method instead of replacing it. Liquid biopsies may not replace tissue biopsies at the present or in the future; but it will let many people to be tested. Furthermore, using a blood test along with tissue will be a breakthrough because, more often than not, the traditional biopsy does not pick enough tissue.^27^,^154,155^

## Conclusion

Taking everything into consideration, liquid biopsy is a new noninvasive sampling tool which brings a new insight into the cancer diagnosis and prognosis perspectives. It can use several components such as cfDNA, CTCs, and exosomes as the real representative of tumor to evaluate the tumor genetic alterations and status. Mostly, for evaluation of the cancer treatment efficacy, the number of CTCs with cutoff 5 per 7.5 mL of blood works. For real-time tracking, the genetic alterations of tumor cfDNA and exosomes can be used to select the best treatment in the way of personalized medicine. In the near future, liquid biopsy will take the place of tissue biopsy or will support the suspicious and nondetermining result of it in an excellent way.
